# Comparison of the Non-VKA Oral Anticoagulants Apixaban, Dabigatran, and Rivaroxaban in the Extended Treatment and Prevention of Venous Thromboembolism: Systematic Review and Network Meta-Analysis

**DOI:** 10.1371/journal.pone.0160064

**Published:** 2016-08-03

**Authors:** A. T. Cohen, M. Hamilton, A. Bird, S. A. Mitchell, S. Li, R. Horblyuk, S. Batson

**Affiliations:** 1 Guy’s and St Thomas’ Hospitals, King’s College, London, United Kingdom; 2 BMS, Princeton, United States of America; 3 Pfizer, Walton Oaks, United Kingdom; 4 Abacus International, Bicester, United Kingdom; 5 Pfizer, New York, United States of America; Kurume University School of Medicine, JAPAN

## Abstract

**Background:**

Historically, warfarin or aspirin have been the recommended therapeutic options for the extended treatment (>3 months) of VTE. Data from Phase III randomised controlled trials (RCTs) are now available for non-VKA oral anticoagulants (NOACs) in this indication. The current systematic review and network meta-analysis (NMA) were conducted to compare the efficacy and safety of anticoagulants for the extended treatment of VTE.

**Methods:**

Electronic databases (accessed July 2014 and updated April 2016) were systematically searched to identify RCTs evaluating apixaban, aspirin, dabigatran, edoxaban, rivaroxaban, and warfarin for the extended treatment of VTE. Eligible studies included adults with an objectively confirmed deep vein thrombosis, pulmonary embolism or both. A fixed-effect Bayesian NMA was conducted, and results were presented as relative risks (RRs). Sensitivity analyses examining (i) the dataset employed according to the time frame for outcome assessment (ii) the model used for the NMA were conducted.

**Results:**

Eleven Phase III RCTs (examining apixaban, aspirin, dabigatran, rivaroxaban, warfarin and placebo) were included. The risk of the composite efficacy outcome (VTE and VTE-related death) was statistically significantly lower with the NOACs and warfarin INR 2.0–3.0 compared with aspirin, with no significant differences between the NOACs. Treatment with apixaban (RR 0.23, 95% CrI 0.10, 0.55) or dabigatran (RR 0.55, 95% Crl 0.43, 0.71) was associated with a statistically significantly reduced risk of ‘major or clinically relevant non-major bleed’ compared with warfarin INR 2.0–3.0. Apixaban also showed a significantly reduced risk compared with dabigatran (RR 0.42, 95% Crl 0.18, 0.97) and rivaroxaban (RR 0.23, 95% Crl 0.09, 0.59). Sensitivity analyses indicate that results were dependent on the dataset, but not on the type of NMA model employed.

**Conclusions:**

Results from the NMA indicate that NOACs are an effective treatment for prevention of VTE or VTE-related death) in the extended treatment setting. However, bleeding risk differs between potential treatments, with apixaban reporting the most favourable profile compared with other NOACs, warfarin INR 2.0–3.0, and aspirin.

## Introduction

Venous thromboembolism (VTE) is a major cause of morbidity and mortality and, consequently, represents a significant burden to global healthcare systems [1, 2]. The condition comprises deep vein thrombosis (DVT) and pulmonary embolism (PE) and has an incidence of approximately 100–200 cases per 100,000 patient-years (148 and 95 per 100,000 patient years for DVT and PE respectively) in six European Union countries [3].

Advances have been made in the diagnosis, treatment, and prevention of VTE, with anticoagulant therapy being the current standard of care. Several risk factors for VTE have also been identified, such as advanced age, surgery, malignancy, trauma and pregnancy [3, 4]. Despite this, the short-term mortality (<6 months after the index event) is approximately 5–10% for DVT and 15–20% for PE [3, 5] and remains high for VTE even 3 years after the event [6]. The burden of VTE is also increased by the risk of further events after the initial episode [7]. Approximately 5–13% of patients with VTE have a recurrence within a year after the index event [6, 8, 9], 25% within 5 years [10] and 30% within 10 years [8].

Current clinical guidelines recommend initial/long-term anticoagulation for up to three months after an initial event [11]. Although the standard of care in this setting was treatment with low molecular weight heparin/warfarin, the non-VKA oral anticoagulants (NOACs) have been shown to be as effective as warfarin in this setting with safety profiles that are comparable or better than LMWH/warfarin [12–19]. A number of meta-analyses and NMAs further support the effectiveness of NOACs within the initial treatment setting [20–24].

The continuation of anticoagulation beyond 3 months is suggested for patients at a high risk of VTE recurrence (i.e. those with an unprovoked event) and an acceptable bleeding risk; patient preference for anticoagulation is also a consideration [11, 25, 26]. Data from Phase III randomised controlled trials (RCTs) in over 7,000 patients are now available on the use of the NOACs in the extended treatment setting (restricted to apixaban, dabigatran, and rivaroxaban) [17–19]. The comparator in the majority of these trials is placebo, as, at the time of patient enrolment, definitive guidance on extended anticoagulation was not available [17–19], with only dabigatran also compared against standard dose warfarin (international normalised ratio (INR) 2.0–3.0) in the RE-MEDY study [18].

However, the relative efficacy and safety of NOACs for extended treatment of VKA is not known, as there are no head-to-head trials. Therefore, the purpose of the current systematic review and network meta-analysis (NMA) is to assess the relative efficacy and safety of NOACs, aspirin, or warfarin (low [INR 1.5–2.0] and standard dose) for the extended treatment and prevention of VTE in adults ≥18 years of age who had received prior treatment for an initial VTE event.

## Methods

### Systematic Review

A systematic review was conducted to identify studies for inclusion in the NMA. Search strings were used to interrogate the following databases: MEDLINE, Embase, and the Cochrane Library (including the Cochrane Database of Systematic Reviews [CDSR], Database of Abstracts of Reviews of Effects [DARE], Cochrane Central Register of Controlled Trials (CENTRAL), Health Technology Assessment Database, NHS Economic Evaluation Database [NHS EED]). Hand searches were conducted for abstracts from conferences of interest (American Society of Haematology [ASH], International Society on Thrombosis and Haemostasis [ISTH], and European Haematology Society [EHS], all from 2011–2013) and reference lists of included articles identified from the electronic searches. The original searches were run on the 14^th^ July 2014 and were updated on the 12^th^ April 2016. The pre-defined inclusion criteria are provided in Table A in S1 File.

Citations were assessed based on title and abstract, and full publications of potentially relevant studies were examined independently by two reviewers using the pre-defined eligibility criteria. Disagreements were resolved via discussion, with a third party if necessary, until a consensus was reached. The quality of RCTs was assessed according to the methodology checklist detailed in Appendix D of the NICE Guidelines Manual 2009 [27].

### Network meta-analysis

In the absence of direct head-to-head comparisons of treatments of interest, NMA is an accepted method of estimating relative treatment effects [28]. WinBUGS software (MRC Biostatistics Unit, Cambridge, UK) was used to conduct a Bayesian fixed-effect (FE) NMA. Due to the small number of studies in the network, the NMA was restricted to a fixed-effect model only. A random-effects model does not provide reliable estimates of the variation in between-study treatment effects when there are few studies in the network of evidence [29]. Indeed, Cochrane guidelines recommend that calculations investigating heterogeneity should be based on a network of at least ten studies [30].

Models were run for 50,000 iterations to calculate the point estimate of comparisons between treatments. The treatment effect was evaluated in terms of relative risk (RR). The point estimate represented the median of the posterior distribution with an associated 95% credible interval (Crl) taken from between the 2.5^th^ and 97.5^th^ percentiles of the distribution of the calculated data. Information on treatment ranking can give misleading results when the evidence network is sparse, and therefore these data are not presented; emphasis is placed on the relative treatment effects and their uncertainty.

### Data sources

The base case analysis was performed on a binary dataset comprising the number at risk and the number of events of an outcome of interest, and used the binomial logit approach to modelling [31]. The primary outcomes of interest were:

1. VTE and VTE-related death. This was a composite efficacy endpoint, consisting of reported events of DVT, fatal or non-fatal PE, and VTE-related death;
2. major bleeding; clinically relevant non-major (CRNM) bleeding; composite of ‘major or CRNM bleeding’;
3. all-cause mortality.

Analyses of efficacy outcomes considered the number of events in the intention-to-treat (ITT) study populations as defined by each study, whereas analyses of bleeding-related outcomes were based on the reported safety populations. The base case analysis employed outcome data reported during intended treatment period.

Additional analyses were conducted for non-fatal PE, DVT, VTE-related death (i.e. death related to any VTE event, or where VTE could not be ruled out as a cause of death), myocardial infarction, and overall treatment discontinuation.

### Sensitivity analyses

Two sensitivity analyses were conducted. In the first, we explored the inclusion of outcome data from the intended follow-up periods of trials. The duration of intended follow-up period (34–38 months) was greater than the duration of intended treatment period (9 months) in WODIT DVT (26) and WODIT PE [32] studies, leading to alternative numbers of outcome events to be used in this sensitivity analysis. This analysis was conducted for the ‘VTE and VTE-related death’ outcome only. This network included the WODIT PE study [32] which was not included in the base-case as data during intended treatment period in the study were not reported. To explore an alternative approach to modelling, a second sensitivity analysis was performed to account for differences in trial follow-up durations. This approach models the outcomes as rate data, using a Poisson log generalised linear approach [31]. The second sensitivity analysis was conducted for the primary outcomes of interest only.

## Results

### Study selection

**Fig 1:**
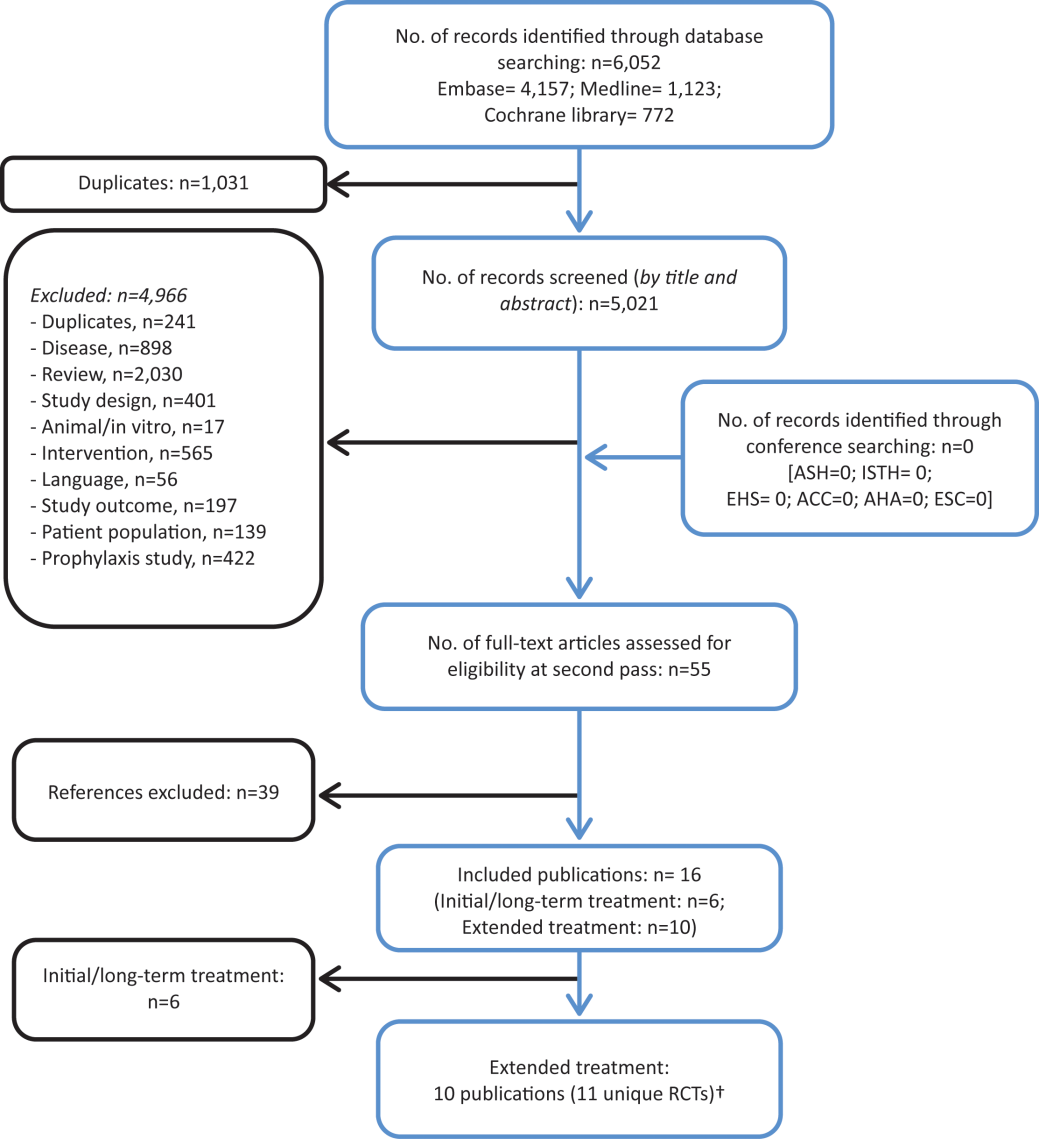
Systematic review flow diagram. The flow diagram indicates inclusion and exclusion of publications at each stage of the systematic review process. †An updated search was conducted in April 2016. No additional eligible publications were identified.

The initial electronic database search identified 6,052 articles, of which 5,021 were screened (after removal of duplicates). In total, 4,966 publications were excluded on the basis of title and abstract. On application of the review inclusion criteria to the 55 full-text papers, a further 39 were excluded. With regards to the updated search conducted in April 2016, 978 citations were screened on the basis of title and abstract. The full text publications of 21 citations were reviewed following exclusion of 957 records, however no additional eligible publications were identified. Therefore, 16 publications met the inclusion criteria and were included in the systematic review (Fig 1).

Six publications reported on initial VTE treatment and are not considered further in the current analysis. Therefore, ten publications reporting on 11 unique trials in the extended treatment of VTE in patients who had received prior initial treatment were included in the NMA: AMPLIFY-EXT[17], EINSTEIN-EXT [19], WARFASA [33], RE-SONATE [18], RE-MEDY[18], ASPIRE [34], LAFIT [35], ELATE [36], WODIT DVT [37], WODIT PE [32], and PREVENT [38]. Due to the inconsistent reporting of data across studies, the number of studies included for the analysis of each outcome varied (Table 1). No data were available for edoxaban in the extended treatment setting.

**Table 1:** Number of studies includes in the NMA for outcomes of interest.

Outcome	No. of included studies
*Base case and sensitivity analysis 1*	
VTE-and VTE related death	10 [17–19, 33–38] †
Major or CRNM bleeding	6 [17–19, 33, 34] †
Major bleeding	11 [17–19, 32–38] †
CRNM bleeding	6 [17–19, 33, 34] †
Mortality	7 [17–19, 34–36, 38]‡
*Sensitivity analysis 2*	
VTE and VTE-related death	11 [17–19, 32–38]†

† Data during intended treatment period not reported in WODIT PE [32]

‡ Data available for RE-MEDY study only [18], not RE-SONATE [18]

### Study characteristics

Table B in S1 File summarises the study details and patient baseline characteristics for each study. The reported mean age of patients and the percentage of female patients were similar across trials. All studies were judged to be of good quality (Table C in S1 File), although there was a mixture of open-label and double-blind studies. Prior treatment duration of studies included in the NMA varied from a minimum of 3 months in the LAFIT [35], ELATE [36], WODIT DVT [37], WODIT PE [32], RE-MEDY [18] and PREVENT [38] trials, up to 6–18 months in the AMPLIFY- EXT [17], EINSTEIN-EXT [19], WARFASA [33] and RE-SONATE [18] trials and 24 months in the ASPIRE trial [34]. The ASPIRE trial included 1% of patients receiving initial anticoagulation for ≤3 months in each arm; however, the overall average duration of initial therapy was ≥6 months [34]. This may suggest a potential variation in the baseline risk of VTE across trials, attributable to differences in the time since the initial VTE event.

The duration of the extended treatment periods also differed between the trials. Duration of intended treatment period ranged from 6 months [18] to 37.2 months [34]. This might lead to variation in the absolute risk of VTE recurrence across trials. Differences between trials with respect to the clinical judgment determining the need for extended anticoagulation after an initial 3 months treatment may have resulted in some heterogeneity of patient risk characteristics across the trials in the NMA. Not all trial publications directly discussed the clinical judgment determining the need for continuation in the design of the trials. Of the studies that reported the proportion of patients with unprovoked VTE, ASPIRE [34], ELATE [36] and AMPLIFY-EXT [17] reported >90% of patients with unprovoked VTE. EINSTEIN-EXT [19] reported 73.7% of patients with unprovoked VTE. Patients with an unprovoked VTE have a higher risk of recurrence [39], suggesting some variation existed in the baseline risk of VTE events between trials. Patients with active cancer are at an increased risk of developing VTE, particularly while receiving systemic chemotherapy [7, 39]. Of the included studies which reported these data, only a small proportion of patients with active cancer were enrolled and, as a consequence, no major impact on the NMA results were expected.

**Fig 2:**
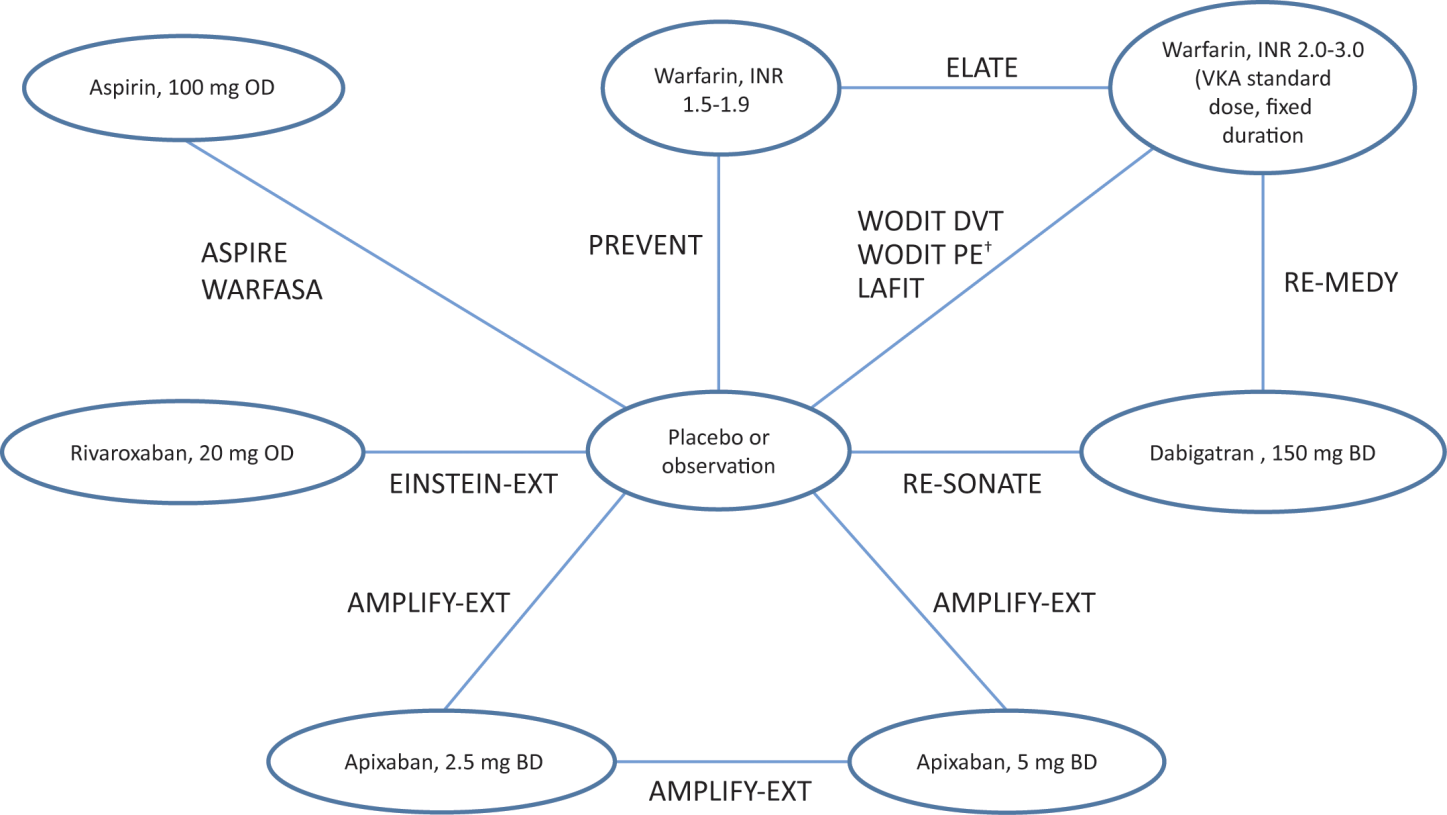
Network of evidence for the primary outcome of ‘VTE and VTE-related death’. † The WODIT PE study was only included in the sensitivity analysis based on outcome data from the intended follow-up period (sensitivity analysis 2).

There may be important differences between definitions used for a particular outcome or the frequency of (or the test used for) outcome assessment. However, a detailed comparison between studies was limited by the degree of reporting in the publications. There was variation in the definition of the primary efficacy outcome across the studies. The ‘VTE and VTE-related death’ endpoint comprised reported events of DVT, fatal or non-fatal PE, and VTE-related death. This was reported in the trial publications as follows: EINSTEIN-EXT [19], PREVENT [38], ELATE [36], WARFASA [33]- recurrent VTE episodes during treatment; LAFIT [35]-VTE; WODIT DVT [37]-risk of VTE recurrence; ASPIRE [34]-recurrent VTE (composite of symptomatic, objectively confirmed DVT, non-fatal PE, or fatal PE); RESONATE [18]- recurrent or fatal VTE or unexplained death; REMEDY [18]- recurrent or fatal VTE. All studies reported a consistent definition of bleeding outcomes as defined by the International Society on Thrombosis and Haemostasis [40]. The network of trials used for ‘VTE and VTE-related death is shown in Fig 2. Additional network diagrams for the primary outcomes of interest are shown in Fig A in S1 File. The raw data used in the NMA (base-case and sensitivity analysis) are presented in Table D in S1 File.

### NMA results

The NMA results for the primary outcomes of interest are presented for the treatment comparisons most commonly used in clinical practice in Table 2.

**Table 2:** Fixed-effect NMA results for primary outcomes of interest using the binomial logit model. Significant results indicated in bold.

Treatment comparison	RR (95% Crl)				
	VTE and VTE-related-death	Major or CRNM bleeding	Major bleeding	CRNM bleeding	Mortality
Apixaban 2.5 mg BD vs. Rivaroxaban 20 mg OD	1.01	**0.23**	**0.03**	**0.28**	1.16
	(0.40, 2.71)	**(0.09, 0.59)**	**(0.00, 0.65)**	**(0.10, 0.73)**	(0.08, 41.59)
Apixaban 2.5 mg BD vs. Dabigatran 150 mg BD	1.77	**0.42**	0.24	0.47	2.17
	(0.70, 4.68)	**(0.18, 0.97)**	(0.02, 1.82)	(0.19, 1.12)	(0.39, 12.56)
Apixaban 2.5 mg BD vs. Aspirin 100 mg OD	**0.28**	0.82	0.34	0.71	0.55
	**(0.14, 0.51)**	(0.33, 2.04)	(0.03, 2.51)	(0.20, 2.43)	(0.17, 1.71)
Apixaban 2.5 mg BD vs. Warfarin INR 2.0–3.0	2.37	**0.23**	**0.13**	**0.26**	1.93
	(0.94, 6.13)	**(0.10, 0.55)**	**(0.01, 0.92)**	**(0.11, 0.64)**	(0.39, 9.91)
Rivaroxaban 20 mg OD vs. Dabigatran 150 mg BD	1.76	1.80	9.00	1.68	1.85
	(0.59, 5.23)	(0.67, 4.83)	(0.50, 151)	(0.62, 4.69)	(0.05, 33.23)
Rivaroxaban 20 mg OD vs. Aspirin 100 mg OD	**0.27**	**3.46**	12.81	2.53	0.47
	**(0.11, 0.59)**	**(1.24, 9.95)**	(0.76, 205)	(0.65, 9.58)	(0.01, 6.05)
Rivaroxaban 20 mg OD vs. Warfarin INR 2.0–3.0	2.34	0.99	4.89	0.93	1.67
	(0.79, 6.76)	(0.37, 2.74)	(0.29, 76.4)	(0.34, 2.66)	(0.04, 27.75)
Dabigatran 150 mg BD vs. Aspirin 100 mg OD	**0.16**	1.93	1.43	1.50	0.25
	**(0.07, 0.34)**	(0.73, 5.07)	(0.32, 6.65)	(0.41, 5.27)	(0.05, 1.22)
Dabigatran 150 mg BD vs. Warfarin INR 2.0–3.0	1.33	**0.55**	0.55	**0.56**	0.89
	(0.76, 2.32)	**(0.43, 0.71)**	(0.28, 1.03)	**(0.42, 0.74)**	(0.45, 1.73)
Aspirin 100 mg OD vs. Warfarin INR 2.0–3.0	**8.60**	**0.29**	0.38	0.37	3.55
	**(4.04, 19.77)**	**(0.11, 0.76**)	(0.09, 1.55)	(0.10, 1.38)	(0.84, 16.20)

Abbreviations: CrI, credible interval; CRNM, clinically relevant non-major; VKA, vitamin K antagonist; VTE, venous thromboembolism.

No statistically significant difference for risk of VTE and VTE-related-death was found for each of the NOACs compared with warfarin INR 2.0–3.0. Apixaban was the only NOAC to show a significantly lower risk of bleeding compared with warfarin INR 2.0–3.0 using all three outcome measures. In contrast, dabigatran showed a statistically significantly lower risk of ‘major or CRNM bleeding’ and CRNM bleeding only compared with warfarin INR 2.0–3.0, and rivaroxaban treatment was associated with no statistically significant differences. With regard to a comparison of the NOACs, apixaban was associated with a statistically significantly lower risk of ‘major or CRNM bleeding’ compared with dabigatran and rivaroxaban. Apixaban also had a statistically significantly lower risk of major bleeding and CRNM bleeding compared with rivaroxaban. There were no other statistically significant differences between the NOACs for the outcomes reported in Table 2, and no statistically significant differences between any treatments in terms of all-cause mortality.

Results for the secondary outcomes of interest (non-fatal PE, DVT, VTE-related death, myocardial infarction, and overall treatment discontinuation) are presented in Table E in S1 File. There were few statistically significant differences between treatments, although the risk of DVT was statistically significantly lower for all NOACs compared with aspirin.

Sensitivity analysis results are reported in Table F in S1 File (sensitivity analysis 1: use of data during intended follow-up period) and Table G in S1 File (sensitivity analysis 2: Poisson model). The use of outcome data from the intended follow-up period of trials for ‘VTE and VTE-related-death’ produced results that were inconsistent with the base-case (using outcome events during intended treatment period): Notably, the direction of treatment effect changed for a number of the comparisons including the comparison of apixaban with warfarin INR 2.0–3.0 which also achieved statistical significance, i.e. the sensitivity analysis showed apixaban to be significantly more efficacious compared with warfarin INR 2.0–3.0 (Table F in S1 File). Results obtained from the Poisson model were broadly in agreement with the base-case analysis, indicating that the choice of model did not impact treatment findings.

## Discussion

Only a small number of large-scale, high quality Phase III RCTs comparing NOACs with VKAs or LMWH have been published to date, and there are no head-to-head comparisons to allow an assessment of the relative efficacy and safety of the NOACs. The current systematic review identified eleven eligible RCTs [12, 17, 18, 32–38] including four RCTs comparing the NOACs with placebo (n = 3) [12, 17, 18] or warfarin INR 2.0–3.0 (dabigatran only) [12, 17, 18, 32–38]. To date, no data for edoxaban have been published in the extended treatment setting.

Several pair-wise meta-analyses examining the NOACs as a class for the extended treatment of VTE have been published [41–44] and results of the analyses indicated that the NOACs had improved efficacy compared with placebo. The current analysis focused on reporting the relative efficacy and safety of the NOACs compared with each other, with warfarin INR 2.0–3.0, and aspirin. In brief, the risk of ‘VTE and VTE-related death’ was statistically significantly lower with the NOACs and warfarin INR 2.0–3.0 compared with aspirin, with no significant differences between the NOACs. Treatment with apixaban or dabigatran was associated with a statistically significantly reduced risk of ‘major or clinically relevant non-major bleed’ compared with warfarin INR 2.0–3.0. Apixaban also showed a significantly reduced risk compared with dabigatran and rivaroxaban.

Four previous NMA publications have reported the relative efficacy and bleeding profile of the individual NOACs for the extended treatment of VTE [45–48], although each analysis was based on a different study network and there was a lack of consistency in the outcomes reported. The analysis by Alotaibi was restricted to an indirect comparison of the three NOAC placebo-controlled RCTs only [12, 17, 18] and reported that the NOACs had a similar odds of VTE recurrence, major bleeding, and mortality compared with each other [48]. Apixaban was reported to be associated with significantly reduced odds of CRNM bleeding compared with both dabigatran and rivaroxaban [48]. The remaining three NMAs [45–47] were based on more extensive networks including both warfarin [45–47] and aspirin [46, 47] and were therefore similar to the network employed in the current analysis. Results from these analyses again confirmed that there was no significant difference between NOACs with regards to the composite outcome of VTE recurrence or all-cause mortality [45–47]. Apixaban was associated with significantly lower risk of major bleeding compared with warfarin INR 2.0–3.0 [45], dabigatran [47], or rivaroxaban [46, 47] and a significantly lower risk of VTE recurrence compared with aspirin 100 mg OD [47].

A number of relative treatment effect estimates from the current NMA for the comparison of NOACs with warfarin were associated with high levels of uncertainty. The relatively small size of the evidence networks (single studies contributing to the majority of direct evidence) and low event numbers (Table D in S1 File) within some of the trials are likely to have contributed to the high levels of uncertainty associated with the estimates of treatment effect. Examples include the comparisons of apixaban and rivaroxaban with warfarin INR 2.0–3.0 for the ‘VTE and VTE-related death’ outcome, which although both comparisons are in favour of warfarin they are associated with large Crls. Fig 2 indicates that the comparison of the two active treatments with warfarin INR 2.0–3.0 is via placebo and the comparison of warfarin INR 2.0–3.0 with placebo is primarily driven by the direct head-to-head data from the WODIT-DVT [37] and LAFIT [35] studies. These studies are the two smallest RCTs in the network in terms of the number of randomised patients, which in turn impacts on the uncertainty of the comparisons of apixaban and rivaroxaban with warfarin INR 2.0–3.0.

The sensitivity analysis based on outcome data from the intended follow-up duration of the trials for the outcome of ‘VTE and VTE-related death’ demonstrates that several of the treatment comparisons are dependent on the dataset used for the analysis. In the sensitivity analysis the main change to the dataset is the event number reported in the WODIT DVT study [37] (Table B in S1 File). The study level treatment effect for the WODIT DVT study based on intended treatment period (base-case) was significantly in favour of warfarin (RR 0.09, 95% CI: 0.01, 0.69). In comparison, the study-level treatment effect indicated equal efficacy between warfarin continuation and warfarin discontinuation when data during the intended follow-up period of the study were used (RR 0.99, 95% CI: 0.57, 1.73). This reduction in the relative efficacy of warfarin treatment in the WODIT DVT study had the effect of increasing the efficacy of active treatments compared with warfarin in the NMA, and indeed the direction of effect was reversed for some treatment comparisons (Table G in S1 File).

The majority of previous NMAs used the binomial approach to statistical modelling. The current NMA is the first to compare the binomial and Poisson (rate) modelling approaches. The similarity of the results of both modelling approaches indicates that differences in time on treatment have no impact on relative treatment effects. Therefore, trials in this indication can be combined in a NMA using a binomial approach despite variation in time on treatment across the studies, as most outcomes of interest occur relatively soon after patient randomisation.

The current NMA confirms the conclusions of the individual trials within the network compared with warfarin INR 2.0–3.0 and shows statistically similar reductions in the risk of VTE or VTE-related death for all NOACs. Compared with dabigatran and rivaroxaban, apixaban was associated with a significantly improved safety profile in terms of a reduction in ‘major or CRNM bleeding’. The use of NOACs, and apixaban in particular, may therefore result in improvements in the extended treatment of VTE [49, 50].

## Supporting Information

S1 File Supporting information. (DOCX)

S2 File PRISMA checklist. (DOC)
