# Correction: Comparison of the Non-VKA Oral Anticoagulants Apixaban, Dabigatran, and Rivaroxaban in the Extended Treatment and Prevention of Venous Thromboembolism: Systematic Review and Network Meta-Analysis

**DOI:** 10.1371/journal.pone.0163386

**Published:** 2016-09-15

**Authors:** A. T. Cohen, M. Hamilton, A. Bird, S. A. Mitchell, X. Li, R. Horblyuk, S. Batson

The fifth authors name is spelled incorrectly. The correct name is Xiaoyan Li. The correct citation is: Cohen AT, Hamilton M, Bird A, Mitchell SA, Li X, Horblyuk R, et al. (2016) Comparison of the Non-VKA Oral Anticoagulants Apixaban, Dabigatran, and Rivaroxaban in the Extended Treatment and Prevention of Venous Thromboembolism: Systematic Review and Network Meta-Analysis. PLoS ONE 11(8): e0160064. doi:10.1371/journal.pone.0160064
