# Supplementary material for: Comparison of the Non-VKA Oral Anticoagulants Apixaban, Dabigatran, and Rivaroxaban in the Extended Treatment and Prevention of Venous Thromboembolism: Systematic Review and Network Meta-Analysis
Source: PLoS One. 2016 Aug 3;11(8):e0160064. doi: 10.1371/journal.pone.0160064 (PMC4972314; doi:10.1371/journal.pone.0160064)
Supplement: S1 File — (DOCX) [file pone.0160064.s001.docx]

**S1 File. Supporting information**

**Figure A. Network diagrams for primary outcomes of interest**

**Table A: Inclusion criteria for the systematic review**

| **Population** | Adult patients (≥18 years of age), who were receiving extended treatment for secondary prevention of VTE † (regardless of length/duration and type of initial therapy) |
| --- | --- |
| **Interventions** | Treatments of interest include:   - NOACs: apixaban, dabigatran, edoxaban, rivaroxaban - Aspirin - Warfarin (standard dose – INR 2.0-3.0; low dose – INR 1.5-1.9) |
| **Comparator** | - Placebo - Warfarin |
| **Outcomes** | Studies were included if they reported ≥ 1 of the following outcomes:  **Efficacy**   - Recurrent VTE and VTE-related death - Non-fatal PE - DVT - VTE-related death - MI - Overall treatment discontinuation - All-cause mortality   **Safety**   - Major and/or CRNM bleeding - Major bleeding - CRNM bleeding - Intracranial bleeding |
| **Study design** | Prospective, phase III RCTs, with no restriction on study design: double-blind or open label |
| **Date and language of publication** | No date restriction; only publications in English language were included |

Abbreviations: CRNM, clinically relevant non-major bleeding; DVT, deep vein thrombosis; INR, international normalised ratio; MI, myocardial infarction; PE, pulmonary embolism; RCT, randomised controlled trial; VKA, vitamin K antagonist; VTE, venous thromboembolism

†The most recent ACCP guidelines ([11](#_ENREF_11)) define extended treatment as anticoagulation continued post 3 months without any scheduled completion date.

**Table B: Summary of study characteristics**

| **Trial** | **Treatment (no. randomised)** | **Mean age (SD)** | **Female,** % | **Idiopathic/ un-provoked VTE by trial %** | **Patients with active cancer, %** | **Index DVT,** % | **Index PE,** % | **Index DVT/PE, %** | **Prior treatment duration** | **Duration of intended treatment period** |
| --- | --- | --- | --- | --- | --- | --- | --- | --- | --- | --- |
| AMPLIFY-EXT ([17](#_ENREF_17))  Double blind, superiority RCT  Multinational | Apixaban 2.5 mg BID (n=840) | 56.6 (15.3) | 42.0 | 91.7 | 1.8 | 64.8 | 35.2 | - | 6-12 months | 12 months |
|  | Apixaban 5 mg BID (n=813) | 56.4 (15.6) | 42.3 |  | 1.1 | 64.8 | 35.2 | - |  |  |
|  | Placebo (n=829) | 57.1 (15.2) | 43.5 |  | 2.2 | 66.5 | 33.5 | - |  |  |
| EINSTEIN-EXT ([12](#_ENREF_12))  Double-blind, inferiority RCT  Multinational | Rivaroxaban 20 mg QD  (n=602) | 58.2 (15.6) | 41.2 | 73.7 | 4.7 | 64.1 | 35.9 | - | 6-12 months | 8.7 months |
|  | Placebo (n=595) | 58.4 (16.0) | 42.9 |  | 4.4 | 60.0 | 40.0 | - |  |  |
| WARFASA ([35](#_ENREF_35))  Double blind RCT  Austria, Italy | Aspirin 100 mg QD (n=205) | 61.9 (15.3) | 34.2 | 100 | NR | 59.5 | 40.5 | - | 6-18 months | 24.0 months |
|  | Placebo (n=198) | 62.1 (15.1) | 38.1 |  | NR | 65.9 | 34.1 | - |  |  |
| RE-SONATE ([18](#_ENREF_18))  Double blind RCT  Multinational | Dabigatran 150 mg BID (n=681) | 56.1 (15.5) | 44.1 | NR | 0 | 63.3 | 26.9 | 6.9 | 6-18 months | 6 months |
|  | Placebo (n=662) | 55.5 (15.1) | 45.0 |  | 0 | 66.6 | 26.9 | 5.3 |  |  |
| RE-MEDY ([18](#_ENREF_18)) ([37](#_ENREF_37)) ([38](#_ENREF_38)) ([40](#_ENREF_40)) ([39](#_ENREF_39))  Double blind RCT  Multinational^†^ | Dabigatran 150 mg BID (n=1,430) | 55.4 (15.0) | 39.1 | NR | 4.2 | 65.6 | 22.7 | 11.7 | 3-12 months | 18 months |
|  | Warfarin INR 2.0-3.0 (n=1,426) | 53.9 (15.3) | 38.9 |  | 4.1 | 64.7 | 23.5 | 11.8 |  |  |
| ASPIRE ([36](#_ENREF_36))  Double blind RCT  Multinational | Aspirin 100 mg QD (n=411) | 55 (16.0) | 45.0 | 95 | 2 | 57.4 | 27.3 | 14.4 | <3 months, 1.1%;  3 to <6 months, 26.2%;  6 to <12 months, 63.8%;  12 to ≤24 months, 9.0% | 37.2 months |
|  | Placebo (n=411) | 54 (15.8) | 46.2 |  |  | 56.5 | 29.0 | 13.6 |  |  |
| LAFIT ([37](#_ENREF_37))  Double blind RCT  Canada, USA | Warfarin INR 2.0-3.0 (n=79) | 59 (16) | 31.6 | 100**^‡‡‡‡^** | 0 | 75.9 | 24.1 | - | 3 months | 10 months |
|  | Placebo (n=83) | 58 (16) | 47.0 |  |  | 73.5 | 26.5 | - |  |  |
| ELATE ([38](#_ENREF_38))  Double blind RCT  Canada, USA | Warfarin INR 1.5-1.9 (n=369) | 57 (16) | 42.5 | 100 | NR | 68.3 | 31.7 | - | ≥3 months | 2.2 years |
|  | Warfarin INR 2.0-3.0 (n=369) | 57 (16) | 47.4 |  |  | 61.5 | 38.5 | - |  |  |
| WODIT DVT ([39](#_ENREF_39))  Open-label RCT  Italy | VKA/  Aceno-coumarol continuation (n=134) | 66.8 (6.7) | 45.5 | 100 | NR | 100 | 0 | - | 3 months | 9 months† |
|  | VKA/  Aceno-coumarol discontinuation / observation (n=133) | 67.7 (7.3) | 38.8 |  |  |  | 0 | - |  |  |
| WODIT PE ([34](#_ENREF_34))  Open-label RCT  Italy | VKA/  Aceno-coumarol continuation (n=165) | 62.9 (16.3) | 60.6 | 55.9 | NR | 0 | 44.8 | 55.2 | 3 months | 9 months‡ |
|  | VKA/  Aceno-coumarol discontinuation / observation (n=161) | 61.0 (15.5) | 58.4 | 56.5 |  | 0 | 44.7 | 55.3 |  |  |
| PREVENT ([40](#_ENREF_40))  Double blind RCT  Canada, USA, Switzerland | Warfarin INR 1.5-1.9 (n=255) | 53 (NR) | 47.1 | 100 | NR | NR | NR | NR | 6.5 months | 2.1 years |
|  | Placebo (n=253) | 53 (NR) | 47.4 |  |  | NR | NR | NR |  |  |

Abbreviations: BD, twice daily; DVT, deep vein thrombosis; LMWH, low molecular weight heparin; NR, not reported; OD, once daily; PE, pulmonary embolism; RCT, randomised controlled trial; SD, standard deviation; VKA, vitamin K antagonist; VTE, venous thromboembolism.

†: Intended follow-up period was 37.8 months (continuation arm)/37.2 months (discontinuation arm). ‡: Intended follow-up period was 33.8 months.

**Table C: Quality assessment of included trials**

| **Question** | **AMPLIFY-EXT (**[**17**](#_ENREF_17)**)** | **EINSTEIN-EXT (**[**12**](#_ENREF_12)**)** | **WARFASA (**[**35**](#_ENREF_35)**)** | **RE-SONATE (**[**18**](#_ENREF_18)**)** | **REMEDY (**[**18**](#_ENREF_18)**)** | **ASPIRE (**[**36**](#_ENREF_36)**)** | **LAFIT (**[**37**](#_ENREF_37)**)** | **ELATE (**[**38**](#_ENREF_38)**)** | **WODIT DVT (**[**39**](#_ENREF_39)**)** | **WODIT PE (**[**34**](#_ENREF_34)**)** | **PREVENT (**[**40**](#_ENREF_40)**)** |
| --- | --- | --- | --- | --- | --- | --- | --- | --- | --- | --- | --- |
| Was randomisation carried out appropriately? | Yes | Yes | Not clear | Yes | Yes | Yes | Yes | Yes | Not clear | Not clear | Yes |
| Was the concealment of treatment allocation adequate? | Not clear | Not clear | Not clear | Not clear | Not clear | Not clear | Not clear | Not clear | Not clear | Not clear | Not clear |
| Were the groups similar at the outset of the study in terms of prognostic factors, for example severity of disease? | Yes | Yes | Yes | Yes | Yes | Yes | Yes | Yes | Yes | Yes | Yes |
| Were the care providers, participants and outcome assessors blind to treatment allocation? If any of these people were not blinded, what might be the likely impact on the risk of bias (for each outcome)? | Yes | Yes | Yes | Yes | Yes | Yes | Yes | Yes | Yes/No | Yes/No | Yes |
| Were there any unexpected imbalances in drop-outs between groups? If so, were they explained or adjusted for? | No | No | No | No | No | No | No | No | No | No | No |
| Is there any evidence to suggest that the authors measured more outcomes than they reported? | No | No | No | No | No | No | No | No | No | No | No |
| Did the analysis include an intention-to-treat analysis? If so, was this appropriate and were appropriate methods used to account for missing data? | Yes (All efficacy analyses included data from the ITT population during the 12-month active study period.  All safety analyses included data from patients during the time they were receiving treatment) | Yes (Primary efficacy analysis was performed on an ITT basis, safety analysis included all patients who received the assigned study drug) | No (The primary efficacy analysis was performed according to a modified ITT principle, with all patients who received at least one dose of the assigned study drug after randomisation included in the analysis) | No (For efficacy, a modified ITT analysis was performed with exclusion of patients who did not receive any dose of the study drug.  All events during the period from receipt of first dose of the study drug until 3 days after receipt of the last dose were included in the safety analysis.) | No (For efficacy, a modified ITT analysis was performed with exclusion of patients who did not receive any dose of the study drug.  All events during the period from receipt of first dose of the study drug until 3 days after receipt of the last dose were included in the safety analysis.  ) | Yes (The two study groups were compared in the primary analysis using an ITT approach, including events up to the final scheduled visit or up to a maximum of 4 years from the time of randomisation. Any patient who stopped using the study drug continued to be followed and was included in the ITT analysis.) | Yes (Main efficacy and safety outcomes were reported for all randomised patients) | Yes (Main efficacy and safety outcomes reported for all randomised patients) | Yes (As some patients discontinued treatment before scheduled completion, continued to use the treatment after scheduled completion, or resumed use of treatment after scheduled interruption, a per protocol analysis was also performed including only the patients who completed treatment according to the study protocol.) | Yes (The primary analysis was performed on an ITT basis) | Yes (The primary analysis was an ITT comparison) |

Abbreviations: ITT, intention-to-treat

**Table D: Raw data used in NMA for primary outcomes of interest**

| **Trial** | **Trial arm** | **VTE and VTE-related-death (base-case†)** | | **VTE and VTE-related-death (sensitivity analysis‡)** | | **Major or CRNM bleeding** | | **Major bleeding** | | **CRNM bleeding** | | **Mortality** | |
| --- | --- | --- | --- | --- | --- | --- | --- | --- | --- | --- | --- | --- | --- |
|  |  | **Number at risk** | **Number of events** | **Number at risk** | **Number of events** | **Number at risk** | **Number of events** | **Number at risk** | **Number of events** | **Number at risk** | **Number of events** | **Number at risk** | **Number of events** |
| AMPLIFY-EXT ([17](#_ENREF_17)) | Apixaban 2.5 mg BD | 840 | 14 | 840 | 14 | 840 | 27 | 840 | 2 | 840 | 25 | 840 | 7 |
|  | Apixaban 5 mg BD | 813 | 14 | 813 | 14 | 811 | 35 | 811 | 1 | 811 | 34 | 813 | 4 |
|  | Placebo | 829 | 73 | 829 | 73 | 826 | 22 | 826 | 4 | 826 | 19 | 829 | 14 |
| EINSTEIN-EXT ([12](#_ENREF_12)) | Rivaroxaban 20 mg OD | 602 | 8 | 602 | 8 | 598 | 36 | 598 | 4 | 598 | 32 | 602 | 1 |
|  | Placebo | 594 | 42 | 594 | 42 | 590 | 7 | 590 | 0 | 590 | 7 | 594 | 2 |
| WARFASA ([35](#_ENREF_35)) | Aspirin 100 mg OD | 205 | 23 | 205 | 23 | 205 | 4 | 205 | 1 | 205 | 3 |  |  |
|  | Placebo | 197 | 39 | 197 | 39 | 197 | 4 | 197 | 1 | 197 | 3 |  |  |
| RE-SONATE ([18](#_ENREF_18)) | Dabigatran 150 mg BD | 681 | 3 | 681 | 3 | 684 | 36 | 684 | 2 | 684 | 34 |  |  |
|  | Placebo | 662 | 37 | 662 | 37 | 659 | 12 | 659 | 0 | 659 | 12 |  |  |
| RE-MEDY ([18](#_ENREF_18)) | Dabigatran 150 mg BD | 1430 | 26 | 1430 | 26 | 1430 | 80 | 1430 | 13 | 1430 | 67 | 1430 | 17 |
|  | Warfarin INR 2.0-3.0 | 1426 | 18 | 1426 | 18 | 1426 | 145 | 1426 | 25 | 1426 | 120 | 1426 | 19 |
| ASPIRE ([36](#_ENREF_36)) | Aspirin 100 mg OD | 411 | 57 | 411 | 57 | 411 | 14 | 411 | 8 | 411 | 6 | 411 | 16 |
|  | Placebo | 411 | 73 | 411 | 73 | 411 | 8 | 411 | 6 | 411 | 2 | 411 | 18 |
| LAFIT ([37](#_ENREF_37)) | Warfarin INR 2.0-3.0 | 79 | 1 | 79 | 1 | NA | NA | 79 | 3 | NA | NA | 79 | 16 |
|  | Placebo | 83 | 17 | 83 | 17 | NA | NA | 83 | 0 | NA | NA | 83 | 8 |
| ELATE ([38](#_ENREF_38)) | Warfarin INR 1.5-1.9 | 369 | 16 | 369 | 16 | NA | NA | 369 | 9 | NA | NA | 369 | 8 |
|  | Warfarin INR 2.0-3.0 | 369 | 6 | 369 | 6 | NA | NA | 369 | 8 | NA | NA | 369 | 1 |
| WODIT DVT ([39](#_ENREF_39)) | VKA continuation | 134 | 1 | 134 | 21 | NA | NA | 134 | 4 | NA | NA | NA |  |
|  | VKA discontinuation / observation | 133 | 11 | 133 | 21 | NA | NA | 133 | 2 | NA | NA | NA |  |
| WODIT PE ([34](#_ENREF_34)) | Warfarin INR 1.5-1.9 | NA | NA | 165 | 15 | NA | NA | 165 | 3 | NA | NA | NA |  |
|  | Placebo | NA | NA | 161 | 18 | NA | NA | 161 | 1 | NA | NA | NA |  |
| PREVENT ([40](#_ENREF_40)) | Warfarin INR 1.5-1.9 | 255 | 14 | 255 | 14 | NA | NA | 255 | 5 | NA | NA | 255 | 4 |
|  | Placebo | 253 | 37 | 253 | 37 | NA | NA | 253 | 2 | NA | NA | 253 | 8 |

Abbreviations: CRNM, clinically relevant non-major; DVT, deep vein thrombosis; MI, myocardial infraction; NA, not applicable ; PE, pulmonary embolism; VTE, venous thromboembolism

† Data from intended treatment period ‡ Data from intended study follow-up period

**Table E: Fixed-effect NMA results for additional outcomes of interest. Significant results indicated in bold.**

| **Treatment comparison** | **RR (95% Crl)** | | | | |
| --- | --- | --- | --- | --- | --- |
|  | **Non-fatal PE** | **DVT** | **VTE-related death** | **Myocardial infarction** | **Overall treatment discontinuation** |
| Apixaban 2.5 mg BD vs. Rivaroxaban 20 mg OD | 3.96  (0.75, 32.91) | 0.70  (0.19, 2.68) | 0.24  (0.01, 12.37) | 0.61  (0.07, 3.96) | 0.72  (0.51, 1.04) |
| Apixaban 2.5 mg BD vs. Dabigatran 150 mg BD | 5.30  (1.01, 46.99) | 1.92  (0.39, 15.39) | 1.16  (0.01, 178) | 0.42  (0.01, 25.20) | 0.78  (0.57, 1.09) |
| Apixaban 2.5 mg BD vs. Aspirin 100 mg OD | NA | **0.12**  **(0.04, 0.29)** | 0.24  (0.01, 12.12) | 1.54  (0.11, 21.41) | **0.66**  **(0.48, 0.91)** |
| Apixaban 2.5 mg BD vs. Warfarin INR 2.0-3.0 | 9.24  (1.51, 95.67) | 2.73  (0.50, 23.42) | 1.13  (0.05, 42.95) | 6.54  (0.06, 1,056) | 0.75  (0.54, 1.05) |
| Rivaroxaban 20 mg OD vs. Dabigatran 150 mg BD | 1.33  (0.12, 14.75) | 2.75  (0.51, 23.46) | 4.97  (0.02, 1,068) | NA | 1.09  (0.76, 1.55) |
| Rivaroxaban 20 mg OD vs. Aspirin 100 mg OD | NA | **0.17**  **(0.05, 0.45)** | 0.99  (0.01, 116) | NA | 0.91  (0.64, 1.31) |
| Rivaroxaban 20 mg OD vs. Warfarin INR 2.0-3.0 | 2.30  (0.20, 29.87) | 3.92  (0.65, 35.42) | 4.91  (0.06, 292) | NA | 1.04  (0.72, 1.50) |
| Dabigatran 150 mg BD vs. Aspirin 100 mg OD | NA | **0.06**  **(0.01, 0.24)** | 0.20  (0.00, 58.0) | 3.62  (0.07, 187) | 0.84  (0.61, 1.17) |
| Dabigatran 150 mg BD vs. Warfarin INR 2.0-3.0 | 1.73  (0.63, 5.11) | 1.41  (0.69, 2.95) | 1.01  (0.03, 37.01) | 13.54  (2.17, 370) | 0.96  (0.82, 1.11) |
| Aspirin 100 mg OD vs. Warfarin INR 2.0-3.0 | NA | 23.57  (5.39, 185) | 5.02  (0.06, 289) | 4.24  (0.04, 651) | 1.21  (0.85, 1.72) |

Abbreviations: CrI, credible interval; CRNM, clinically relevant non-major; NA, not applicable; VKA, vitamin K antagonist; VTE, venous thromboembolism.

**Table F: Sensitivity analysis 1. Fixed-effect NMA results for ‘VTE and VTE-related death’ using outcome data reported during the intended follow-up period of trials. Significant results indicated in bold.**

| **Treatment comparison** | **RR (95% Crl)** | |
| --- | --- | --- |
|  | **Data from intended treatment period** | **Data from intended follow-up period** |
| Apixaban 2.5 mg BD vs. Rivaroxaban 20 mg OD | 1.01  (0.40, 2.71) | 1.01  (0.39, 2.72) |
| Apixaban 2.5 mg BD vs. Dabigatran 150 mg BD | 1.77  (0.70, 4.68) | 0.61  (0.28, 1.30) |
| Apixaban 2.5 mg BD vs. Aspirin 100 mg OD | **0.28**  **(0.14, 0.51)** | **0.27**  **(0.14, 0.50)** |
| Apixaban 2.5 mg BD vs. Warfarin INR 2.0-3.0 | 2.37  (0.94, 6.13) | **0.49**  **(0.24, 0.95)** |
| Rivaroxaban 20 mg OD vs. Dabigatran 150 mg BD | 1.76  (0.59, 5.23) | 0.61  (0.23, 1.46) |
| Rivaroxaban 20 mg OD vs. Aspirin 100 mg OD | **0.27**  **(0.11, 0.59)** | **0.27**  **(0.11, 0.57)** |
| Rivaroxaban 20 mg OD vs. Warfarin INR 2.0-3.0 | 2.34  (0.79, 6.76) | 0.49  (0.20, 1.09) |
| Dabigatran 150 mg BD vs. Aspirin 100 mg OD | **0.16**  **(0.07, 0.34)** | **0.44**  **(0.24, 0.78)** |
| Dabigatran 150 mg BD vs. Warfarin INR 2.0-3.0 | 1.33  (0.76, 2.32) | 0.80  (0.49, 1.28) |
| Aspirin 100 mg OD vs. Warfarin INR 2.0-3.0 | **8.60**  **(4.04, 19.77)** | **1.82**  **(1.14, 2.95)** |

Abbreviations: CrI, credible interval; CRNM, clinically relevant non-major; VKA, vitamin K antagonist; VTE, venous thromboembolism.

**Table G: Sensitivity analysis 2. Fixed-effect NMA results for primary outcomes of interest using the Poisson modelling approach. Significant results indicated in bold.**

| **Treatment comparison** | **HR (95% Crl)** | | | | |
| --- | --- | --- | --- | --- | --- |
|  | **VTE and VTE-related-death** | **Major or CRNM bleeding** | **Major bleeding** | **CRNM bleeding** | **Mortality** |
| Apixaban 2.5 mg BD vs. Rivaroxaban 20 mg OD | 0.83  (0.33, 2.27) | **0.19**  **(0.06, 0.49)** | **0.18**  **(0.02, 1.05)** | **0.22**  **(0.08, 0.61)** | 0.94  (0.06, 33.98) |
| Apixaban 2.5 mg BD vs. Dabigatran 150 mg BD | 1.85  (0.72, 4.94) | **0.40**  **(0.16, 0.94)** | **0.21**  **(0.03, 0.88)** | 0.47  (0.19, 1.15) | 2.39  (0.43, 14.20) |
| Apixaban 2.5 mg BD vs. Aspirin 100 mg OD | **0.27**  **(0.14, 0.49)** | 0.81  (0.31, 2.09) | 0.33  (0.04, 1.38) | 0.73  (0.19, 2.53) | 0.54  (0.16, 1.67) |
| Apixaban 2.5 mg BD vs. Warfarin INR 2.0-3.0 | 2.46  (0.98, 6.33) | **0.22**  **(0.08, 0.53)** | **0.09**  **(0.01, 0.36)** | **0.26**  **(0.10, 0.67)** | 2.13  (0.44, 10.94) |
| Rivaroxaban 20 mg OD vs. Dabigatran 150 mg BD | 2.24  (0.74, 6.62) | 2.09  (0.74, 6.67) | 1.15  (0.29, 4.21) | 2.12  (0.76, 6.51) | 2.53  (0.06, 48.24) |
| Rivaroxaban 20 mg OD vs. Aspirin 100 mg OD | **0.32**  **(0.13, 0.69)** | **4.30**  **(1.44, 13.76)** | 1.80  (0.46, 6.84) | 3.26  (0.78, 13.74) | 0.57  (0.02, 7.90) |
| Rivaroxaban 20 mg OD vs. Warfarin INR 2.0-3.0 | **2.97**  **(1.00, 8.50)** | 1.14  (0.39, 3.71) | 0.48  (0.13, 1.78) | 1.18  (0.40, 3.76) | 2.25  (0.06, 39.25) |
| Dabigatran 150 mg BD vs. Aspirin 100 mg OD | **0.14 (0.06, 0.31)** | 2.03  (0.74, 5.73) | 1.54  (0.63, 4.02) | 1.54  (0.39, 5.67) | 0.23  (0.04, 1.07) |
| Dabigatran 150 mg BD vs. Warfarin INR 2.0-3.0 | 1.32  (0.76, 2.33) | **0.55**  **(0.41, 0.72)** | **0.42**  **(0.25, 0.72)** | **0.56**  **(0.41, 0.74)** | 0.89  (0.45, 1.74) |
| Aspirin 100 mg OD vs. Warfarin INR 2.0-3.0 | **9.26**  **(4.35, 20.99)** | **0.27**  **(0.09, 0.76)** | **0.27**  **(0.12, 0.64)** | 0.36  (0.10, 1.44) | 3.93  (0.97, 18.01) |

Abbreviations: CrI, credible interval; CRNM, clinically relevant non-major; ; NA, not applicable; VKA, vitamin K antagonist; VTE, venous thromboembolism.
